# Successful Treatment with Lurasidone of First-Episode Psychosis in Down Syndrome: Case Report and Literature Review

**DOI:** 10.1192/j.eurpsy.2022.2065

**Published:** 2022-09-01

**Authors:** L. Delgado Montfort

**Affiliations:** Parc Taulí University Hospital, Mental Health, Sabadell, Spain

**Keywords:** antipsychotic drugs, Down syndrome, Psychosis, pharmacological treatment

## Abstract

**Introduction:**

Co-morbid psychiatric disorders are common in Down syndrome (DS). Evidence is limited for pharmacotherapy, specifically antipsychotics, for psychiatric co-morbidity in DS.

**Objectives:**

To describe a case of a patient with DS who developed a first-episode psychosis (FEP) and who responded to lurasidone in monotherapy and to review recent literature on the treatment of psychosis in patients with DS.

**Methods:**

(1) Case report: FEP in DS patient treated with lurasidone 37 mg/day. (2) Narrative review on the treatment of psychosis in DS patients through PubMed database (1990-2020). Key terms: “psychosis”, “Down Syndrome”, “pharmacological treatment”, “antipsychotic drugs”.

**Results:**

A 21-year-old woman with DS, without psychiatric history, presenting with behavioural anomalies, aggressiveness, soliloquies, and unmotivated laughs was referred to our outpatient clinic by her general practitioner. Symptoms began one year prior and progressively worsened, impairing her daily functionality. Previous blood workup was normal. She was diagnosed with FEP and began treatment with lurasidone 37 mg. At 4-week follow-up, she showed total remission of the psychotic symptoms, had no tolerability complaints, and returned to baseline functionality levels. Discussion: No reports of lurasidone use in psychosis in DS have been published. To treat psychotic symptoms in DS, most literature reports describe the use of typical antipsychotics, which are usually effective, but often poorly tolerated; atypical antipsychotics such as risperidone and aripiprazole have also been used.

**Conclusions:**

Lurasidone may be a useful option in patients with FEP in DS. Further research is warranted on treatment of psychosis in this population.

**Disclosure:**

No significant relationships.

